# Cancer Risk and Behavioral Factors, Comorbidities, and Functional Status in the US Elderly Population

**DOI:** 10.5402/2011/415790

**Published:** 2011-07-12

**Authors:** Igor Akushevich, Julia Kravchenko, Lucy Akushevich, Svetlana Ukraintseva, Konstantin Arbeev, Anatoliy Yashin

**Affiliations:** ^1^Center for Population Health and Aging, Duke University, Durham, NC 27708, USA; ^2^Duke Cancer Institute, Duke University, Durham, NC 27705, USA

## Abstract

About 80% of all cancers are diagnosed in the elderly and up to 75% of cancers are associated with behavioral factors. An approach to estimate the contribution of various measurable factors, including behavior/lifestyle, to cancer risk in the US elderly population is presented. The nationally representative National Long-Term Care Survey (NLTCS) data were used for measuring functional status and behavioral factors in the US elderly population (65+), and Medicare Claims files linked to each person from the NLTCS were used for estimating cancer incidence. The associations (i.e., relative risks) of selected factors with risks of breast, prostate, lung and colon cancers were evaluated and discussed. Behavioral risk factors significantly affected cancer risks in the US elderly. The most influential of potentially preventable risk factors can be detected with this approach using NLTCS-Medicare linked dataset and for further deeper analyses employing other datasets with detailed risk factors description.

## 1. Introduction

About 80% of all cancers are diagnosed at ages above 65 years, and up to 75% of cancers are thought to be associated with behavioral factors—if modified, they could significantly reduce cancer burden [1]. Analyzing an impact of the modifiable factors on cancer risk, it has been speculated that about 50% of cancers are potentially preventable [2]. Although there are many specific results clarifying the effects of lifestyle factors on risk of lung, breast, prostate, colorectal, and other cancers, both the roles of various lifestyle factors and combined effects of multiple factors are still not clear. The availability of large datasets with more detailed information provides a new prospective in studying the role of behavioral factors in the cancer risk both for each factor alone and by taking into account risk factor interactions. 

The sources for obtaining the evidence on associations between behavioral factors and cancer risk include *in vitro* studies, animal experiments, ecological studies, and case-control studies. However, there are certain limitations in providing with exposure-to-a-factor—cancer risk correlations [3, 4]. The most influential is study design biases (e.g., selection bias): for example, due to the fact that information on behavioral factors is usually collected by interviewing the patients with diagnosed cancers thus causing the bias of the estimates. The prospective cohort studies can avoid most of methodological biases; however, they are typically expensive, especially, when detailed questionnaires are required. 

In this paper, analysis of multiple associations between behavior factors and cancer risk is presented using the National Long-term Care Survey linked to Medicare files of service use. The developed approach is free of many limitations usually accompanying similar studies. First, our approach is based on the cohort study in which the measurements were performed before the beginning of the cohort followup for cancer incidence. Therefore, selection or recall biases which are typical for case-control studies are not the case in the study design. Second, in earlier studies, the evaluated associations of the same type, and especially relative risks obtained for different lifestyle factors could hardly be compared between each other due to the differences in the study designs, time of measurements, and so forth. In contrast, data used in our study included multiple risk factors which were measured simultaneously, thus providing with possibility to compare evaluated associations between different risk factors. The used dataset is useful for both getting an additional knowledge about the roles of already recognized cancer risk factors as well as to establish new candidate behavioral factors which could potentially influence cancer risk in the US elderly which will provide with the background hypothesis to be tested in further analyses. Third, this study is based on population which is representative of the whole US elderly population, thus allowing to overcome such limitations of meta-analysis as heterogeneity bias, publication bias, and several others (reviewed by Manton, Akushevich, and Kravchenko [5], Sections 3.1 and 3.2).

## 2. Data and Methods

Two sources of data are used in the analysis: the nationally representative NLTCS, for measuring functional status and behavioral factors in the elderly, and Medicare Claims linked to the NLTCS, for cancer incidence in the US population. Breast, prostate, lung, and colon cancers were selected for analyses due to their high-incidence rates in elderly and because their incidence rates can be relatively well reconstructed from Medicare data. The SEER data were used as “a gold standard” to compare age patterns of selected cancers from SEER with age patterns prediction based on NLTCS-Medicare data. 

### 2.1. Medicare Claims Data

Medicare is the primary health insurer of 97% of the U.S. population aged 65+ years. All Medicare beneficiaries receive Part A benefits, which cover inpatient care in short- and long-stay hospitals, skilled nursing facilities, home health, and hospice care. About 95% of beneficiaries are also subscribed to Medicare Part B to obtain the benefits covering physician service, outpatient care, durable medical equipment, and home health (in certain cases). The Medicare claims records contain the information on dates and costs of each service, types of providers, ICD-9-CM diagnoses, auxiliary diagnostic codes, and procedure codes. 

### 2.2. The National Long-Term Care Survey

The NLTCS (1982, 1984, 1989, 1994, 1999, and 2004/5) contains longitudinal and cross-sectional data on a nationally representative sample of about 49,000 US individuals aged 65+ years, with 17,000–20,000 age-eligible survivors in each of six rounds. The 1994 and 1999 NLTCS waves were analyzed: more than 200 variables were selected in each wave of survey being grouped as follows (a complete list of all variables used in the analysis is presented in Table 1 in the Electronic Supplementary Material available online at doi: 10.5402/2011/415790):

- demographic characteristics (4 variables: sex, race, marital status, and urban versus rural living);
- self-reported comorbidity (27 major medical conditions and recent medical problems);
- daily living activities (22 variables: 6 activities of daily living (ADLs) with the two severity levels and 10 instrumental activities of daily living (IADLs));
- range of motion (16 variables reflecting ability to perform daily activities such as walking, using fingers to grasp and handle small objects, climbing stairs);
- physical activity list (29 variables, 25 of them reflecting specific physical activities (e.g., golf, tennis) were measured in 1994 only);
- nutrition and social activities (30 variables, 24 of them representing a nutrition survey were measured in 1999 only);
- alcohol consumption and smoking (4 variables, reflecting two severity levels);
- other functioning (28 variables reflecting self-estimates of health, information about mood, habits, keeping in touch with friends and relatives, and if they are satisfied with their life);
- housing and neighborhood characteristics (23 variables describing the area, housing, and amenities where sample lives, as well as including information whether he lives with other household members, and neighborhood characteristics);
- health insurance (6 variables containing information on coverage by Medicare, HMO, Medicaid, etc.);
- medical providers and prescription medicine (44 variables providing with information on the use of health care services and public and private expenditures for health care services);
- cognitive functioning (18 variables about cognitive status of individuals, 10 of them are measured in 1994 and 11 of them are measured in 1999);
- income and assets (4 variables correlated with socioeconomic status of individuals);
- body mass index (5 variables representing Body-mass index and eating style).

The following concept was used for selecting the variables to be studied. First, we collected all substantive variables measured in certain NLTCS surveys which were independent from responses to other questions. The most of variables were binary, and the variables with multiple outcomes were dichotomized by aggregating outcomes with similar meanings. Then, among these variables only those with low frequencies for missing data were kept: the frequencies for missing data were less than 0.02 for 65% of variables, 0.02–0.05: for 16% of variables, 0.05–0.15: for 8% variables, 0.15–0.25: for 8% of variables, and 0.25–0.45: for 2% of variables (the variables of the last group contain the questions about individual's cognitive status). 

### 2.3. Methods for Association Studies

For each variable (232:for 1994 and 229:for 1999 surveys) the association with four cancers incidence were analyzed using 1994 and 1999 surveys, in total, 3,688 associations. The empirical analysis and methods of univariate, two-factor, and multivariate statistical estimation with Cox's proportional hazards model were used, with individual weights (so-called, CDS Detailed Cross-Sectional NLTCS weights) for obtaining the US elderly population relevant results. The standard errors for all estimates were calculated based on real numbers of individuals, that is, for nonweighted populations. For small numbers of individuals in a certain stratum, corrections for the standard calculation of standard errors were used according to [6]. 

For lung, colon, breast, and prostate cancers, the age-adjusted incidence rates conditional on a specific outcome (i.e., specific answer on a specific question) were estimated and the relative risks were estimated as the ratios of the rates for alternative outcomes. Note, that age-adjusted risks were calculated for subpopulations with different responses for certain questions/variables (e.g., current smokers and nonsmokers) using the same population weights for both outcomes. Therefore, the rates conditional on a specific outcome of each variable were adjusted for total population, thus taking into account a possible effect of age dependence of certain outcome prevalence. For example, lung cancer rates in smokers and nonsmokers were adjusted for age structure of total population to include smokers, non-smokers, and individuals with missing information on smoking status. 

Calculations of relative risks of specific outcomes for all cancers were also performed in the univariate proportional hazard model. SAS software PHREG was used for parameter estimation. Two basic methods of individual followups were used and compared: (1) the time-period-based followup started from the date of individual interview for which stratification by age and sex were used at the maximization of partial likelihood, and (2) the age-based followup started from the age at interview. 

### 2.4. Procedure for Onset Identification

The age at onset was defined for each studied cancer. First, individual medical histories were reconstructed from all Medicare files combining all records with respective ICD-9 codes: breast cancer (174.xx), prostate cancer (185.xx), lung cancer (162.xx), and colon cancer (153.xx). Second, individuals with the histories of the considered cancer before the date of interview were excluded. Then, the date of Medicare record (referred as “this record” below in this subsection) was identified with the date of cancer onset if both two below conditions are satisfied:

- this record was the earliest record with respective ICD code as a primary diagnosis in one of four Medicare sources (inpatient care, outpatient care, physician services, and skilled nursing facilities);
- there was another record with respective ICD code as a primary diagnosis in these four Medicare sources which appeared in another claim and on a date other than the date of this record and no later than 0.3 of a year after this record.

Since we analyzed the cases starting from 1994 and Medicare histories were available from 1991, we had a sufficient time period (>36 months) to reject the prevalent cases. In this analysis we also excluded the individuals with additional coverage by HMO, as well as individuals enrolled into Medicare less than half a year before the interview in year 1994 or 1999. Table 1 presents the age-adjusted rates of cancer incidence in 1994–1998 and 1999–2004 compared with those calculated for these periods using SEER data.

## 3. Results and Discussion

Age-adjusted estimates for associations between behavioral factors and the risk of four most common cancers (lung, prostate, breast, and colon) were calculated by three methods: (i) calculating the age-adjusted rates with adjusting of each subpopulation (i.e., with positive and negative outcomes for a specific question) for total population; (ii) based on the proportional hazard model with two approaches for choosing the follow-up variables based on age and (iii) on time. All three methods took into account the fact that age is the main and well-documented cancer risk factor. They were designed to analyze the associations for same-age individuals. 

### 3.1. Associations with Cancer Risk: Results and Discussion

Using the three approaches discussed above, we calculated the age-adjusted associations between behavioral factors and the risk of four most common cancers (lung, prostate, breast, and colon) and selected the most significant lifestyle variables associated with increased cancer risk. A specific list of selected associations depends on selection criteria. The strictest criterion used in the analysis was based on the Bonferroni correction and an additional requirement that the found associations have to be detected both in the cohort of 1994 and 1999. Only two associations were found to satisfy this criterion: heavy cigarette smoking and lung cancer (RR = 7), and cancer history presence (cancer site nonspecified) and breast cancer risk (RR = 6) (in part, the high relative risk could be due to a mixture of the prevalence cases which cannot be separated from incident cases). The Bonferroni correction is too conservative, and it is supposed to be applied to independent hypotheses testing, which is not the case of this study due to the explanatory variables correlated especially within the specific groups. Therefore, two other criteria based on *P*-values equaling  .05 and  .002 are used. 

Keeping the associations with *P* < .05 detected at least by two of three methods resulted in a list containing 40 variables for breast cancer, 25 for prostate cancer, 43 for lung cancer, and 23 for colon cancer (see Table 2 in the Electronic Supplementary Material available online at doi: 10.5402/2011/415790). Tables 2(a)–2(d) shows the sublist that included variables for which at least one of six relative risks estimated by three methods for two years has *P*-value lower than  .002 (marked with bold font). The majority of these variables were obtained from the subgroups such as comorbidity and health status, housing and neighborhood characteristics, nutrition, social activities, and other functioning. Specific variables in the list from the other groups are body mass index and the type of insurance coverage for breast and lung cancers, alcohol consumption and physical activity for prostate and colon cancers, and cigarette smoking for lung cancer. 

Comorbidity is an important risk factor of cancer mortality, however its role in cancer risk is not so clear and varies depending on cancer site. Our results demonstrated that the comorbidity effect was larger for breast and colon cancers and less pronounced (but still significant) for lung cancer. Specifically, circulatory disease and certain neurological disorders increased breast cancer risk, and pulmonary diseases were associated with increased risks of lung and colon cancers. For example, having pneumonia during last year was associated with increased risk of colon cancer (RR = 2.9), and emphysema was associated with the increased lung cancer risk (RR = 3). It has been shown in other studies that prior history of respiratory diseases such as emphysema, asthma, and pneumonia was associated with increased lung cancer risk, for example, for emphysema OR = 2.87 [7]. The causal nature of the association between respiratory diseases and lung cancer remains speculative because since both emphysema and chronic bronchitis are strongly influenced by smoking. There is an evidence that inflammation may also play a role in colon carcinogenesis (through C-reactive protein and, probably, interleukin-6 factors), however, epidemiological studies are sparse [8]. Also pneumonia could be associated with smoking, which in turn may increase the risk of colon neoplasia [9]. At present, however, there is no proven hypothesis about the role of respiratory diseases in lung and colon carcinogenesis, and empirical evidences are not entirely consistent and are largely derived from the observational epidemiologic studies [10]. 

For prostate cancer, there were indications of inverse association with comorbidity in our study, for example, prostate cancer risk was lower for persons with arthritis (both osteo- and rheumatoid arthritis) (RR = 0.48). The inverse associations for other comorbidities were not significant; however they did demonstrate the tendency. Our study also demonstrated the reduced prostate cancer risk (RR = 0.45) in patients with self-reported diabetes. These results are in agreement with recently published data from the Prostate Cancer Prevention Trial in which diabetes was associated with reduced risk of prostate cancer: OR = 0.53 and OR = 0.72 were detected of the risk of a low-grade and high-grade tumors, respectively.  Particularly significant inverse association with prostate cancer risk (OR = 0.27) was found for early-onset diabetes (diagnosed before age 30) [11, 12]. However, the mechanisms underlying these associations are still not completely clear. 

We have found that physical activity decreased risks of all four studied cancers, with a more significant decrease in individuals who reported moderate activities (RR = 3.5). The effect of vigorous activities was also positive (i.e., reducing cancer risk); however, the estimates of RRs varied depending on the type of physical activity. Note, that while analyzing the effects of physical activity the bias could occur due to the difficulties in measuring this factor, its overreporting, and confounding factors. However, the inverse associations with physical activity (i.e., reducing cancer risk) have been described in other studies for most of human cancers, including colorectal, breast, prostate, and lung [13]. 

Our results demonstrated that maintaining normal body weight was associated with decreased risks of cancers of breast (RR = 0.55), prostate (RR = 0.6), and colon (RR = 0.4). A “tradeoff” between the effects of BMI (measured, not self-reported) on breast and lung cancer risks was detected: while normal BMI (18–25 kg/m^2^) reduced breast cancer risk twice, lung cancer risk increased more than three times, and vice versa, that is, BMI above 25 kg/m^2^ doubled breast cancer risk while diminished risk of lung cancer. The inverse association between BMI and lung cancer could be due to confounding smoking (i.e., smokers may maintain lower BMI easier). Data from the multiple case-control and cohort studies suggest this possibility: after adjustment for confounding smoking, the inverse association became insignificant [14]. Other studies showed that an excessive calorie intake was strongly related to colon and postmenopausal breast cancer risk [15]; however, not enough evidence was provided for prostate cancer risk of its association with body mass index [14]. 

Two variables were used in our study to characterize alcohol consumption: (i) “drinking alcoholic beverages such as beer, wine, or liquor no more than 1–3-times a month or not drinking at all”, and (ii) consuming the alcohol “at least 1 or 2 times a week”. No significant associations were found for the first variable, while the second variable (i.e., heavier alcohol consumption) was associated with increased prostate cancer risk (RR = 1.7). This is in agreement with recent results obtained from the Prostate Cancer Prevention Trial [16]. Besides, we did not consider cancers for which alcohol consumption an evident risk factor.

The effects of dietary patterns on cancer risk in our study were not statistically significant for all studied cancers, and associations for these cancers were also not proved in other studies [17–20]. Specifically, no clear association was found between fruits and vegetables consumption and reduced colon cancer risk. This is in agreement with weak or nonsignificant associations obtained from other studies. These results do not support the existence of protective role of dietary fiber against colon cancer [21, 22]. Also, no association was found in our study between beef, pork, and lamb (without specification on well-done or other cooking regimen) consumption and increased colon cancer risk. The recent meta-analyses showed that high intake of processed meat but not fresh meat could increase risk of colon cancer [23, 24], while well-done meat could increase colon cancer risk in susceptible individuals with rapid-rapid phenotypes of NAT2 and CYP1A2 [25]. 

No associations have been found in our study between breast, colon, and lung cancer risk and different levels of disability. This is in accordance with the results obtained from other studies where no significant protective effects of disability were found [26]. However, a markedly decreased risk of breast cancer was observed among disabled older women compared with physically capable but inactive women [27]. In our study the positive association between decreased risk and disability was detected for prostate cancer. 

Several stable associations, which cannot be straightforwardly interpreted, were detected. These associations are of noncausal character and can be further investigated in two-factor analysis using other measured variables as confounders or mediators for their explanation. They could also be due to unobserved heterogeneity in cancer risk and could potentially be clarified in future studies. An example of such an association is the relationship between breast cancer risk and nine variables from the HNC group (e.g., positive responses to questions like “Which of these things would make things easier or more comfortable for you: extra wide doors or hallways, push bars on the door, extra handrails, etc?”) which are strongly associated with breast cancer risk with RR from 4 to 7 and *P*-value of the association less than  .001. No associations of these variables were found for risks of other cancers. 

Because of occurrence of false-positive results while testing the hypotheses and/or noncausal nature due to observed and unobserved confounding, the second step in the analysis was the two-factor analysis including effects of interactions between risk factors allowing for revealing effects of confounding and effectively taking into account the mutual correlations inside the groups of similar questions.

### 3.2. Two-Factor Analysis

Analysis of simultaneous effects of two variables allowed us to check whether associations found in unidimensional analyses were confounded by other measured variables. Simultaneous effects of all possible pairs of variables were evaluated using the Cox proportional model focusing on detecting the significant change in the estimated relative risk (or the loss of the significance of the estimate) after adding the second variable. Several types of the effects of second variables were identified: confoundings, candidate mediators, independent predictors, and overlapping predictors (notation is discussed by [28, 29]). 

As expected, smoking was the strongest and most often confounding of other risk factors for lung cancer: it changed substantially the effects of sex (by 2.2 *σ*_*E*_, where *σ*_*E*_ is the standard error of the RR estimate in unidimensional analysis, in 1994 and by 0.6 *σ*_*E*_ in 1999), urban living (0.8 *σ*_*E*_), easy loosing temper (1.2 *σ*_*E*_), emphysema (0.7 *σ*_*E*_), and BMI (0.7 *σ*_*E*_) on lung cancer risk. The BMI changed effects of diet (about 1.0 *σ*_*E*_) and lost appetite (0.6 *σ*_*E*_). Smoking, physical activity, social activity, satisfaction with life, overeating, type of medical insurance, and access to medical services (except of the Veteran Administration insurance) were independent from mediating lung cancer risk by other factors. Certain variables whose effects changed the initial effect of independent variable (i.e., that in unidimensional analysis) can be causally linked to the effect of independent variable; therefore, they can be considered as mediators. Detailed analysis of such causal pathways requires further investigation using a theory of statistical mediation MacKinnon [29] and will be performed elsewhere. 

For other cancers, the confounding/mediation effects were less noticeable. The BMI influenced effects of certain comorbidities (such as circulatory diseases, about 1.0 *σ*_*E*_) and overeating (0.6 *σ*_*E*_) on breast cancer risk, while the effects of vitamins, social activities/hobbies, and contacts with relatives were independent from the effects of other. For colon cancer, BMI, being almost an independent factor, mediated the effects of sex (1.4 *σ*_*E*_), alcohol consumption (0.8 *σ*_*E*_), and overeating (0.7 *σ*_*E*_). 

The two-factor analysis also revealed the situations with overlapping effects among variables which are correlated, codominant, and have no temporal precedence, for example, effect of variables from the HNC group on breast cancer risk. This is because of their mutual correlation and the so-called effect of statistical collinearity, when the estimated effect of a predictor cannot be interpreted itself. Summarizing, the effects of confounding evaluated in two-factor analysis do not change the conclusions made while using univariate approach but further specify the evaluated associations. Also this analysis clarified that further progress can be achieved by investigating the combined effect of correlated variables (e.g., those from the same NLTCS group) by constructing an aggregated index.

## 4. Outlook and Conclusion

In this study, we analyzed how lifestyle factors represented by a number of variables were associated with incidence of four most prevalent cancers such as lung, prostate, breast, and colon. Overall view on the results of association analyses allowed us to describe population groups of higher and lower risks of these cancers. Being a smoker was the main characteristic of elderly population group of higher risks of lung cancer, with comorbidity (e.g., emphysema), lower BMI, and poor functional status also each playing the role. The population of higher risk of colon cancer was characterized by a higher BMI and comorbidity. The elderly women at higher breast cancer risk reported higher occasional activity and intentions to improve things around her day to day (these relationships could be indirect and require further investigation). The group of higher risk of prostate cancer had lower comorbidity, disability, and functional status (partly, it could be due to the underdiagnoses in individuals with poor health state). 

In this study, many well-recognized associations were confirmed; however, certain fundamental questions about lifestyle effects on cancer incidence remain unclear. Specifically, directions for further investigations could include analyses (i) of comorbidity variables for which the inverse associations with prostate cancer was found (e.g., arthritis or diabetes), (ii) of associations indirectly related to cancer risk variables (e.g., housing, neighborhood, or income characteristics) which could be potentially explained in terms of confounding factors. From biomedical perspective, potential extension of this study could include the NLTCS-Medicare data analysis clarifying (i) how found cancer site-specific associations could be affected by racial disparities and (ii) whether there is a difference in factors effects and their mediation for cancers of reproductive and nonreproductive systems as well as more detailed analysis of sex-specific associations and potentially different role of certain factors in mortality and cancer risk in males and females.

Further analysis could deal with implementation of interactions between two or more variables using multi-factor analysis, applying the theory of statistical mediation [29], searching for so-called instrumental variables, and constructing quasirandomization using propensity score approach (reviewed by Faries et al. [30]). For example, the propensity score can be evaluated for each association by considering respective independent variable as “exposure” or “treatment” and using all other measured variables as predictors of the “exposure” in logistic regression. Another further investigation could focus on searching the latent variables capable of describing the heterogeneity in cancer risk: for example, for lung cancer such a variable could be self-care, psychological condition, or happiness (the latter is also a candidate variable for breast cancer). One formal method capable of identification of the latent variables associated with certain risk is the linear latent structure analysis [31, 32] when a score is identified by statistical methods and analyzed in a certain basis each component of which is associated with a group of higher/lower risk. Important feature of the method is that it takes into account mutual correlation between predictors.

The most influential (i.e., demonstrated the strongest association with cancer risk) of potentially controllable risk factors can be detected using the approach developed in this paper and then applied to further deeper analyses, including other data sets with detailed risk factors description/characteristics, for example, analyses of duration of exposure and intensity of risk factor. These approaches could provide with the steps toward the individualized forecasting of cancer risk potentially resulting in preventive strategies which could be oriented to population groups with specific characteristics such as those obtained from the indices and association/confounding findings of this study.

## Supplementary Material

Table 1 of Electronic Supplementary Material. List of all variables used and their outcomes in 1994 and 1999 surveys. Relative risks are given in Tables 2 of Electronic Supplementary Material and in Table 2 in the text.
Table 2 of Electronic Supplementary Material. Relative risks (only significant estimates are shown) of incidental cancer for specific variables, four cancer sites, and two surveys (1994 and 1999). The values of relative risks correspond to outcomes in column ‘outcome'. Three used methods are marked as AA (age-adjusted), CT (time follow-up in Cox model), and CA (age follow-up in Cox model).

## Figures and Tables

**Table 1:** One year rate of cancer incidence per 100,000.

Cancer site	Breast		Prostate		Lung		Colon	
Year	1994	1999	1994	1999	1994	1999	1994	1999
Rate from NLTCS	529	472	960	802	337	327	216	199
Rate from SEER	448	439	991	964	345	338	232	218

**Table:** (a) Female breast cancer

*N*	Variable	RR94 AA	RR94 CT	RR94 CA	RR99 AA	RR99 CT	RR99 CA
B10	Has no other cancers being diagnosed	**0.26**	**0.19**	**0.22**	**0.14**	**0.12**	**0.09**
B12	Has no insomnia/frequent trouble sleeping	—	—	—	0.47	**0.22**	0.46
B18	Has no hypertension in the last year	**0.34**	**0.43**	**0.33**	—	—	—
D13	Needs hearing aid	1.98	—	—	**3.39**	—	**4.52**
F1	Does not visit senior center regularly	—	—	—	**0.13**	**0.16**	**0.14**
F6	Does not take vitamins/supplements	—	—	—	**0.16**	0.31	0.17
H17	Did not read magazine/newspaper in the last week	**0.11**	0.15	—	—	—	—
H18	Did not work on hobby in the last week	0.5	**0.39**	0.48	—	—	—
H25	Did not lose appetite during the last two weeks	0.38	**0.29**	**0.3**	—	—	—
I2	Does not live in community for retired or disabled persons	—	—	—	**0.28**	—	**0.25**
I4	Lives in house/apartment with ramps	—	—	—	**5.72**	—	**5.1**
I5	Lives in house/apartment with elevator	—	—	—	**6.63**	—	**6.64**
I6	Lives in house/apartment with extra wide door	—	—	—	3.31	2.66	**5.58**
I8	Lives in house/apartment with raised toilet	—	—	—	**5.66**	3.15	**6.42**
I10	Thinks that *grab bars* would make things more comfortable	—	—	—	**5.2**	3.2	**5.87**
I11	Thinks that *ramps* would make things more comfortable	—	—	—	**8.13**	**5.75**	**16.19**
I12	Thinks that *elevators or stair lifts* would make things more comfortable	—	—	—	**5.31**	**5.61**	**16**
I13	Thinks that *extra wide door* would make things more comfortable	—	—	—	**6.44**	4.5	**15.55**
I14	Thinks that *push bars on doors* would make things more comfortable	—	—	—	**7.07**	**14.12**	**30**
I15	Thinks that *raised toilet *would make things more comfortable	**3.55**	**5.4**	**5.89**	**3.72**	**4.56**	**8.92**
I16	Does not think that *any above item * make things more comfortable	**0.41**	**0.33**	**0.38**	**0.33**	0.45	**0.25**
J6	Is not covered by private health insurance plan	**0.16**	0.24	0.19	—	—	—
K3	Did not stay in a hospital overnight in the last year	—	—	—	**0.29**	**0.21**	**0.23**
K15	Did not get optometrist care in the last month	—	—	—	2.39	**4.35**	—
K38	Household Members will end up paying for medical services	3.72	3.32	**5.28**	**7.59**	—	5.07
K39	Children or non-household members will end up paying for medical services	—	—	—	**7.55**	—	4.52
M4	Lives in house/apartment	0.42	**0.31**	0.39	—	—	—

**Table:** (b) Prostate cancer

*N*	Variable	RR94 AA	RR94 CT	RR94 CA	RR99 AA	RR99 CT	RR99 CA
B1	Has no rheumatism/arthritis	—	—	—	**2.41**	2.21	—
B20	Has no arms/legs circulation troubles in the last year	**7.05**	3.65	4.85	**3.36**	—	—
C5	ADL: cannot bath	**0.23**	—	—	**0.16**	—	—
C6	ADL: cannot use toilet	0.09	—	—	**0.09**	—	—
C10	IADL: cannot prepare meal	0.17	—	—	**0.1**	—	—
C11	IADL: cannot shoppe for groceries	**0.17**	—	—	**0.23**	—	—
D3	Very difficult to climb one flight of stairs	0.34	—	—	**0.08**	—	—
F10	Often eats eggs	—	—	—	0.48	0.46	**0.44**
F17	Often eats pasta such as spaghetti or noodles	—	—	—	**0.33**	0.49	**0.34**
G3	Consumes alcohol at least once per week	**2.17**	2.01	1.79	—	—	—
H12	Nobody checks/calls to make sure he/she is all right	**3.21**	2.44	3.34	2.05	—	—
H17	Did not read magazine/newspaper in the last week	0.34	—	—	**0.18**	—	—
H19	Did not play games (e.g., solitaire) or work on puzzles in the last week	—	—	—	2.33	2.87	**3.12**
H22	Did not attend civic/religious/other meetings in the last month	—	—	—	**0.43**	0.58	0.52
K18	Did not receive medical care in doctor's office in the last month	1.76	—	1.77	**4.52**	2.62	**4.02**
K44	Has bought more than 2 prescription medicine in the last month	0.52	0.57	0.58	**0.33**	0.54	0.42

**Table:** (c) Lung cancer

*N*	Variable	RR94 AA	RR94 CT	RR94 CA	RR99 AA	RR99 CT	RR99 CA
A1	Male	**2.86**	2.13	**3.01**	1.73	—	1.8
B24	Did not have emphysema in the last year	—	—	—	**0.34**	0.32	**0.27**
B27	Did not have broken other than hip bones in the last year	**0.35**	—	—	**0.26**	—	0.37
D7	Very difficult reach above head	**2.6**	—	—	**3.12**	—	2.58
D10	Very difficult grasp and handle small objects	**4.53**	—	3.57	—	—	—
E10	Did bowling in the past 2 weeks	5.16	**6.07**	**6.22**	—	—	—
F1	Does not visit senior center regularly	**0.33**	0.45	0.46	—	—	—
F11	Often eats poultry	—	—	—	**0.38**	—	0.44
G1	Current non-smoker	**0.21**	**0.24**	**0.2**	0.45	0.38	**0.33**
G4	Smokes at least 1 pack per day	**7.39**	**5.77**	**6.51**	**7.04**	**7.19**	**8.3**
H8	Does not lose her/his temper	0.42	**0.3**	0.39	—	—	—
H11	Does not forget to eat, take medicine, and pay bills	—	—	—	**0.3**	0.36	**0.31**
H17	Did not read magazine/newspaper in the last week	**2.26**	1.95	2.1	0.47	—	—
H27	Not satisfied with his/her life as a whole	**3.42**	—	3.36	—	—	—
I5	Lives in house/apartment with elevator	**5.48**	—	**4.49**	—	—	—
I20	No food or grocery store in the neighborhood	—	—	—	**2.57**	2.2	2.28
J4	Is not covered by public assistance program paying for health care	0.26	0.16	**0.12**	—	—	—
J6	Is not covered by private health insurance plan	—	—	—	**3.11**	—	**2.6**
K14	Did not receive care from podiatrist in last month	—	—	—	**3.64**	2.89	**3.63**
K20	Does not have a regular source of medical care	**3.42**	2.71	**3.22**	—	—	—
K36	Medicaid will end up paying for medical services	**3.41**	—	—	2.23	—	2.91
K37	Veterans Administration will end up paying for medical services	**4.83**	—	3.63	—	—	—
M4	Lives in house/apartment	—	—	—	**0.35**	—	0.35
N2	Has normal BMI (18–25 kg/m^2^)	—	—	—	**3.33**	3.08	**3.63**
N3	Has BMI >25 kg/m^2^	—	—	—	0.3	0.34	**0.28**

**Table:** (d) Colon cancer

*N*	Variable	RR94 AA	RR94 CT	RR94 CA	RR99 AA	RR99 CT	RR99 CA
B2	Does not have paralysis	**0.16**	0.29	0.22	**0.25**	—	—
B16	Did not have heart attack in the last year	—	—	—	**0.25**	—	0.22
B17	Did not have other heart problems in the last year	—	—	—	**0.34**	—	0.3
D15	Uses devices other than glasses/lenses, hearing aid, and artificial larynx	**5.31**	—	**4.37**	—	—	—
E1	Walked for exercise in the past 2 weeks	**2.64**	1.83	2.54	—	—	—
E29	Often drinks coffee/tea	—	—	—	0.42	0.39	**0.27**
H17	Did not read magazine/newspaper in the last week	1.89	—	—	**2.78**	—	—
I3	Lives in house/apartment with grab bars	**2.45**	1.88	2.12	—	—	—
I13	Thinks that *extra wide door* would make things more comfortable	**3.91**	3.51	—	—	—	—
K3	Did not stay in hospital overnight in the last year	—	—	—	0.46	**0.3**	0.38
K35	Medicare will end up paying for medical services	3.4	**4.21**	**5.03**	—	—	—
L15	Cannot tell what is the name of this city	—	—	—	**10.93**	—	18.73
