# Supplementary material for: Cancer Risk and Behavioral Factors, Comorbidities, and Functional Status in the US Elderly Population
Source: ISRN Oncol. 2011 Jul 12;2011:415790. doi: 10.5402/2011/415790 (PMC3197174; doi:10.5402/2011/415790)
Supplement: Supplementary file 1 — Table 1 of Electronic Supplementary Material. List of all variables used and their outcomes in 1994 and 1999 surveys. Relative risks are given in Tables 2 of Electronic Supplementary Material and in Table 2 in the text. Table 2 of Electronic Supplementary Material. Relative risks (only significant estimates are shown) of incidental cancer for specific variables, four cancer sites, and two surveys (1994 and 1999). The values of relative risks correspond to outcomes in column ‘outcome'. Three used methods are marked as AA (age-adjusted), CT (time follow-up in Cox model), and CA (age follow-up in Cox model). [file 415790.f1.pdf]

Table 1 of Electronic Supplemental Material. List of all variables used and their outcomes in 1994 and 1999 surveys. Relative risks are given in Tables 2 of Electronic Supplementary Material and in Table 2 in the text.

	Variable	1994	1999
A	DEMOGRAPHIC CHARACTERISTICS (Dem)		
A1	Sex	Male	Male
A2	Race	nonwhite	nonwhite
A3	Marital status	not married (check)	not married (check)
A4	Urban/Rural	city	city
B	CONDITION LIST (CND):		
B1	Rheumatism or arthritis	No	No
B2	Paralysis	No	No
B3	Other permanent numbness or stiffness (besides paralysis, rheumatism or arthritis)	No	No
B4	Multiple sclerosis	No	No
B5	Cerebral palsy	No	No
B6	Epilepsy	No	No
B7	Parkinson's disease	No	No
B8	Glaucoma	No	No
B9	Diabetes	No	No
B10	Cancer	No	No
B11	Frequent Constipation	No	No
B12	Frequent trouble sleeping	No	No
B13	Frequent severe headaches	No	No
B14	Obesity or he/she is overweight	No	No
B15	Arteriosclerosis or hardening of the arteries of the arteries	No	No
B16	Has he/she had any of the following in the last 12 months: A heart attack	No	No
B17	Has he/she had any of the following in the last 12 months: Any other hear problem	No	No
B18	Has he/she had any of the following in the last 12 months: Hypertension or high blood pressure	No	No
B19	Has he/she had any of the following in the last 12 months: A stroke	No	No
B20	Has he/she had any of the following in the last 12 months: Circulation trouble in his/her arms or legs	No	No
B21	Has he/she had any of the following in the last 12 months: Pneumonia	No	No
B22	Has he/she had any of the following in the last 12 months: Bronchitis	No	No
B23	Has he/she had any of the following in the last 12 months: Flu	No	No
B24	Has he/she had any of the following in the last 12 months: Emphysema	No	No
B25	Has he/she had any of the following in the last 12 months: Asthma	No	No
B26	Has he/she had any of the following in the last 12 months: A broken hip	No	No
B27	Has he/she had any of the following in the last 12 months: Other broken bones	No	No
C	Disability: ADL/IADL (ADL)		
C1	ADL eating	can't	can't
C2	ADL getting in/out of bed	can't	can't
C3	ADL getting around inside	can't	can't
C4	ADL dressing	can't	can't
C5	ADL bathing	can't	can't
C6	ADL getting to bathroom/using toilet	can't	can't
C7	IADL doing heavy work	can't	can't
C8	IADL doing light work	can't	can't
C9	IADL doing laundry	can't	can't
C10	IADL preparing meals	can't	can't
C11	IADL shopping for groceries	can't	can't
C12	IADL getting around outside	can't	can't
C13	IADL going places outside of walking	can't	can't
C14	IADL managing money	can't	can't
C15	IADL taking medicine	can't	can't
C16	IADL making telephone calls	can't	can't
C17	ADL cannot eat at all	can't	can't
C18	ADL cannot get out of bed at all	can't	can't

Table 1 of Electronic Supplemental Material. List of all variables used and their outcomes in 1994 and 1999 surveys. Relative risks are given in Tables 2 of Electronic Supplementary Material and in Table 2 in the text.

	Variable	1994	1999
C19	ADL cannot get around inside at all	can't	can't
C20	ADL cannot dress at all	can't	can't
C21	ADL did not bath at all	can't	can't
C22	ADL did not get to bathroom/using toilet at all	can't	can't
D	RANGE OF MOTION (RMI)		
D1	Is he/she missing any fingers, a hand, or an arm		No
D2	Is he/she missing any toes, a foot, or a leg	No	No
D3	How difficult is it for him/her to: climb one flight of stairs	very difficult/can't do	very difficult/can't do
D4	How difficult is it for him/her to: walk to the end of a room and back	very difficult/can't do	very difficult/can't do
D5	How difficult is it for him/her to: bend to put on socks or stockings	very difficult/can't do	very difficult/can't do
D6	How difficult is it for him/her to: lift a 10-pound package like a bag of groceries and hold it for a few minutes	very difficult/can't do	very difficult/can't do
D7	How difficult is it for him/her to: reach above head	very difficult/can't do	very difficult/can't do
D8	How difficult is it for him/her to: comb or brush hair	very difficult/can't do	very difficult/can't do
D9	How difficult is it for hin/her to: wash hair	very difficult/can't do	very difficult/can't do
D10	How difficult is it for him/her to: use fingers to grasp and handle small objects	very difficult/can't do	very difficult/can't do
D11	Does he/she usually see well enough to read ordinary newsprint, with or without glasses or contact lenses	No	No
D12	Which of these devices does he/she uses: glasses/contact lenses	use	use
D13	Which of these devices does he/she uses: hearing aid	use	use
D14	Which of these devices does he/she uses: artificial larynx	use	use
D15	Which of these devices does he/she uses: other	use	use
D16	Which of these devices does he/she uses: none	not used	not used
E	ACTIVITY LIST (AL)		
E1	In the past 2 weeks did he/she do: walking for exercise	yes	
E2	In the past 2 weeks did he/she do: jogging or running	yes	
E3	In the past 2 weeks did he/she do: hiking	yes	
E4	In the past 2 weeks did he/she do: gardening or yard work	yes	
E5	In the past 2 weeks did he/she do: aerobics or aerobic dancing	yes	
E6	In the past 2 weeks did he/she do: other dancing	yes	
E7	In the past 2 weeks did he/she do: calisthenics or general exercise	yes	
E8	In the past 2 weeks did he/she do: golf	yes	
E9	In the past 2 weeks did he/she do: tennis	yes	
E10	In the past 2 weeks did he/she do: bowling	yes	
E11	In the past 2 weeks did he/she do: biking	yes	
E12	In the past 2 weeks did he/she do: swimming or water exercise	yes	
E13	In the past 2 weeks did he/she do: yoga	yes	
E14	In the past 2 weeks did he/she do: weight lifting or training	yes	
E15	In the past 2 weeks did he/she do: basketball	yes	
E16	In the past 2 weeks did he/she do: baseball or softball	yes	
E17	In the past 2 weeks did he/she do: football	yes	
E18	In the past 2 weeks did he/she do: soccer	yes	
E19	In the past 2 weeks did he/she do: volleyball	yes	
E20	In the past 2 weeks did he/she do: handball, racquetball, or squash	yes	
E21	In the past 2 weeks did he/she do: skating	yes	
E22	In the past 2 weeks did he/she do: skiing	yes	
E23	In the past 2 weeks did he/she do: any other physically active hobbies	yes	
E24	In the past 2 weeks did he/she do: none	not at all	
E25	Does he/she exercises or plays sports regularly	No	
E26	Would he/she say that he/she is physically more, less, or about as active as other persons your age	Less active/same	
E27	On a usual day, how much time does he/she spend on vigorous activities	More than 10 min	More than 10 min
E28	On a usual day, how much time does he/she spend on moderate activities	More than 10 min	More than 10 min
E29	On a usual day, how much time does he/she spend on light activities	More than 10 min	More than 10 min

Table 1 of Electronic Supplemental Material. List of all variables used and their outcomes in 1994 and 1999 surveys. Relative risks are given in Tables 2 of Electronic Supplementary Material and in Table 2 in the text.

	Variable	1994	1999
F	NUTRITION & SOCIAL ACTIVITIES (NSA)		
F1	Does he/she regularly go to a senior center	No	No
F2	Does he/she regularly go to an adult day care center	No	No
F3	Does he/she receive any health services or therapy at the center	No	No
F4	Does this center provide him/her with transportation between the center and his/her home	No	No
F5	Does he/she now regularly eat meals in a senior center or some other place with a special meal for older people	No	No
F6	Does he/she usually take a vitamin and/or mineral supplement once a week or more	No	No
	How often during the past three months he/she has consumed several foods by themselves or as a part of mixed dishes:		
F7	Lowfat milk?		often
F8	Whole milk?		often
F9	Other dairy products such as yogurt or cheese?		often
F10	Eggs?		often
F11	Poultry such as chicken or turkey?		often
F12	Beef, pork, or lamb?		often
F13	Processed meats such as frankfurters or luncheon meats?		often
F14	Fish or shellfish?		often
F15	Breads?		often
F16	Rice and other grains such as barley or oats?		often
F17	Pasta such as spaghetti or noodles?		often
F18	Pizza?		often
F19	Potatoes?		often
F20	Fortified breakfast cereals?		often
F21	Other breakfast cereals?		often
F22	Dried peas, beans, or other legumes?		often
F23	Vegetables?		often
F24	Fruits and juices?		often
F25	Butter, margarine, mayonnaise, or salad dressing?		often
F26	Sweet baked goods such as cookies or sweet rolls?		often
F27	Candy?		often
F28	Sugared soft drinks?		often
F29	Coffee or tea?		often
F30	Alcohol, including liquor, beer, and wine?		often
G	ALCOHOL CONSUMPTION & SMOKING (ACS)		
G1	Does he/she currently drink any kind of alcoholic beverage, such as beer, wine, or liquor	No	No
G2	Does he/she currently smoke	No	No
G3	Combined Variabe: Intensity of alcohol consumption (at least 1 time a week)	yes	yes
G4	Combined Variabe: Intensity of smoking (at least 1 pack per day)	yes	yes
H	OTHER FUNCTIONING (OFN)		
H1	Compared to other persons the same age, would he/she say that his/her health is…	fair/poor	fair/poor
H2	How often does he/she avoid doing things because he/she does not have enough energy to do them	rarely	rarely
H3	Does he/she ever feel he/she needs the help of a doctor or counselor for a mental or emotional problem	No	No
H4	Was he/she ever hospitalized for a mental/emotional problem	No	No
H5	Was he/she hospitalized for a mental or emotional problem in the last 5 years	No	No
H6	Has a doctor ever advised him/her either recently or a long time ago--to get treatment for a mental or emotional problem	No	No
H7	Has a doctor told him/her that (listed above) in the past 5 years	No	No
H8	Does he/she lose your temper and throw, kick, slam, or destroy things…	not at all	not at all
	In the past month did he/she at any time...		
H9	...lose way and not find the way back	no	no

Table 1 of Electronic Supplemental Material. List of all variables used and their outcomes in 1994 and 1999 surveys. Relative risks are given in Tables 2 of Electronic Supplementary Material and in Table 2 in the text.

	Variable	1994	1999
H10	...take any money or anything else that didn? belong to ...without realizing it_	no	no
H11	...forget to do important things like eat, take medicine, or pay...bills	no	no
H12	Does anyone phone or check on him/her regularly just to make sure he.she is all right	no	no
H13	Does he/she keep in touch with any relatives either by visiting or by phone	no	no
H14	Does he/she keep in touch with any friends, including neighbors he/she considers as friends, either by visiting or by telephone	no	no
H15	Sometimes people feel that they have nobody to tell their troubles to. Would he/she say he/she feels this way	rarely	rarely
H16	Does he/she have any pets	no	no
H17	During the past week, did he/she read a book, magazine, or newspaper	no	no
H18	During the past week, did he/she work on a hobby, like painting, sewing, or arts and crafts	no	no
H19	During the past week, did he/she play games such as solitaire or work on puzzles	no	no
H20	During the past week, did he/she listen to records, tapes, (or CDs--in 1994)	no	no
H21	During the past month did he/she go to religious services	no	no
H22	During the past month did he/she attend a meeting of a civic, religious, professional, or recreational club or org.	no	no
H23	During last two weeks did not feel like doing the things he/she usually does	no	no
H24	During last two weeks could not sleep like he/she usually does	no	no
H25	During last two weeks lost his/her appetite / could not eat like he/she usually does	no	no
H26	Taken all together, how would he/she say things are these days…	not too happy	not too happy
H27	Generally speaking, how satisfied is he/she with his/her life as a whole…	not satisfied	not satisfied
H28	About how many hours a day does he/she usually watch television	more than 1 hour	more than 1 hour
I	HOUSING AND NEIGHBORHOOD CHARACTERISTICS (HNC)		
I1	All things considered, how satisfied is he/she with the place in which he/she is living…	not satisfied	not satisfied
I2	Is this place part of a building or community intended for older or retired, or disabled persons	no	no
	Which of these things does he/she have in...(house/apartment)		
I3	… extra handrails or grab bars	has	has
I4	... ramps	has	has
I5	… elevators or stair lifts	has	has
I6	… extra wide doors or hallways	has	has
I7	… push bars on doors	has	has
I8	… raised toilet	has	has
I9	… none	none	none
	Which of these things would make things easier or more comfortable for him/her		
I10	… extra handrails or grab bars	has	has
I11	… ramps	has	has
I12	… elevators or stair lifts	has	has
I13	… extra wide doors or hallways	has	has
I14	… push bars on doors	has	has
I15	… raised toilet	has	has
I16	… none	none	none
I17	Is there a toilet or portable toilet conveniently located in the room in which he/she sleeps	no	no
I18	Is there a toilet or portable toilet conveniently located in the room in which he/she spends most of his/her day	no	no
I19	All things considered, how satisfied is he/she with this neighborhood…	not satisfied	not satisfied
I20	Is there a conveniently located food or grocery store in the neighborhood	no	no
I21	Is there a conveniently located drug store in the neighborhood	no	no
I22	Is crime a serious problem in this neighborhood	no	no
I23	During the past year, has he/she (or any members of his/her household) been a victim of a crime in this neighborhood	no	no
J	HEALTH INSURANCE (INS)		
J1	Is he/she now covered by Medicare	no	no
J2	During the past 12 months, has he/she received health care which has been or will be paid for by Medicaid	no	no

Table 1 of Electronic Supplemental Material. List of all variables used and their outcomes in 1994 and 1999 surveys. Relative risks are given in Tables 2 of Electronic Supplementary Material and in Table 2 in the text.

	Variable	1994	1999
J3	Does he/she now have a Medicaid card	no	no
J4	Is he/she now covered by any other public assistance program that pays for health care	no	no
J5	Is he/she now covered by Champus or Champva...	no	no
J6	Is he/she now covered by a private health insurance plan which pays any part of a hospital, doctor's, or surgeon's bill	no	no
K	MEDICAL PROVIDERS AND PRESCRIPTION MEDICINES (MPP)		
K1	Has he/she ever been a patient in a nursing home, convalescent or rest home	no	no
K2	Is he/she now on a waiting list to go into a nursing home	no	no
K3	Has he/she been a patient in a hospital overnight or longer, in the last 12 months	no	no
K4	In the last month...did he/she see a physical therapist, an occupational therapist, or a speech, or a hearing therapist (not counting when he/she was in the hospital)	no	no
K5	Who will end up paying: fee for service insurance plans	marked	marked
K6	Who will end up paying: HMO/prepaid group	marked	marked
K7	Who will end up paying: Medicare	marked	marked
K8	Who will end up paying: Medicaid	marked	marked
K9	Who will end up paying: Veterans Administration	marked	marked
K10	Who will end up paying: Household Members	marked	marked
K11	In the last month...did he/she discuss any personal problems with a psychiatrist, psychologist, or any other mental health professional	No	No
K12	In the last month...did he/she receive care from a dentist, foot doctor, optometrist, or chiropractor	No	No
K13	...did he/she receive care from a Dentist in last month	marked	marked
K14	...did he/she receive care from a Foot doctor in last month	marked	marked
K15	...did he/she receive care from a Optometrist in last month	marked	marked
K16	...did he/she receive care from a Chiropractor in last month	marked	marked
K17	In the last month...did he/she go to an emergency room or hospital clinic when he/she did not stay overnight.	No	No
K18	(Not counting any visits mentioned above)...did he/she receive medical care in a doctor's office	No	No
K19	In the last month...did he/she see a doctor in his/her home...	No	No
K20	Does he/she have a regular source of medical care, like a family doctor, a clinic, or some other medical person or place	No	No
K21	In the last month...did he/she receive nursing services at home from someone such as a visiting nurse, home health aide, or nurse's aide	No	No
K22	In the last month, did he/she have any health problem or condition about which he/she would have liked to see a doctor or other medical person, but did not	No	No
	What is the reason that he/she did not see a doctor or other medical person:		
K23	... financial	marked	marked
K24	... time	marked	marked
K25	... availability of a doctor	marked	marked
K26	... transportation	marked	marked
K27	... not free to leave	marked	marked
K28	... problem not serious	marked	marked
K29	... afraid to find out what's wrong	marked	marked
K30	... weather	marked	marked
K31	... other	marked	marked
K32	Will insurance, Medicare, Medicaid, or anyone else, including any members of his/her family, end up paying any of the charges for those visits	No	No
K33	Who will end up paying: Fee for service insurance plans	marked	marked
K34	Who will end up paying: HMO/prepaid group	marked	marked
K35	Who will end up paying: Medicare	marked	marked
K36	Who will end up paying: Medicaid	marked	marked
K37	Who will end up paying: Veterans Administration	marked	marked
K38	Who will end up paying: Household Members	marked	marked
K39	Who will end up paying: Children of sample person (non-household members)	marked	marked
K40	Who will end up paying: other	marked	marked
K41	Who will end up paying: Public Program (non-Medicaid)	marked	marked
K42	Who will end up paying: Private Charity	marked	marked

Table 1 of Electronic Supplemental Material. List of all variables used and their outcomes in 1994 and 1999 surveys. Relative risks are given in Tables 2 of Electronic Supplementary Material and in Table 2 in the text.

	Variable	1994	1999
K43	Is he/she taking any prescription medicine to help calm him/her down or to relieve depression	No	No
K44	In the last month...how many prescription medicines were bought or obtained for him/her	>2+ medicine	>2+ medicine
L	COGNITIVE FUNCTIONING (CF)		
L1	What is the date today	not correct	not correct
L2	What day of the week is this	not correct	not correct
L3	What is your street address	not correct	not correct
L4	In what state is this	not correct	
L5	How old are you	not correct	
L6	When were you born	not correct	
L7	Who is the President of the United States now	not correct	
L8	Who was the President just before him	not correct	
L9	What was your mother's maiden name	not correct	
L10	Subtract 3 from 20 and keep subtracting 3 from each new	not correct	
L11	What is the name of this month?		not correct
L12	What year is it?		not correct
L13	What time of the year is it?		not correct
L14	What is the name of this country?		not correct
L15	What is the name of this city?		not correct
L16	What are the names of the two streets in the closest intersection?		not correct
L17	What floor are we on now?		not correct
L18	Please repeat these three objects.		all answers are correct
M	INCOME AND ASSETS (INC)		
M1	Did he/she or any members of his/her family who live here receive food stamps in the last month	no	no
M2	Did he/she (or any member of his/her family who live here) receive any other welfare payments…	no	no
M3	Which of these types of places is he/she living in now	Alone/with others in house/apartment (independent living)	Alone/with others in house/apartment (independent living)
M4	What are the living quarters?	House, apartment of flat (vs room or mobile home)	House, apartment of flat (vs room or mobile home)
N	Body Mass Index (BMI)		
N1	Combined Variable: Body Mass Index: Low	lower than 18 kg/m**2	lower than 18 kg/m**2
N2	Combined Variable: Body Mass Index: Normal	18-25 kg/m**2	18-25 kg/m**2
N3	Combined Variable: Body Mass Index: High	higher than 25 kg/m**2	higher than 25 kg/m**2
N4	On a typical day, how many meals Does he/she eat	>3 times	>3 times
N5	Besides breakfast lunch and dinner how many other times during the day does he/she eat.	>+1 times	>+1 times
