# Supplementary material for: Cancer Risk and Behavioral Factors, Comorbidities, and Functional Status in the US Elderly Population
Source: ISRN Oncol. 2011 Jul 12;2011:415790. doi: 10.5402/2011/415790 (PMC3197174; doi:10.5402/2011/415790)
Supplement: Supplementary file 2 [file 415790.f2.pdf]

Table 2 of Electronic Supplemental Material. Relative risks (only significant estimates are shown) of incidental cancer for specific variables, four cancer sites, and two surveys (1994 and 1999). The values of relative risks correspond to outcomes in column 'outcome'. Three used methods are marked as AA (age-adjusted), CT (time follow-up in Cox model), and CA (age follow-up in Cox model).

	Variable	Outcome	Cancer	RR94, AA	RR94, CT	RR94, CA	RR99, AA	RR99, CT	RR99, CA
B10	Cancer	No	Breast	**0.26**	**0.19**	**0.22**	**0.14**	**0.12**	**0.09**
B12	Frequent trouble sleeping	No	Breast	.	.	.	0.47	**0.22**	0.46
B14	Obesity or he/she is overweight	No	Breast	0.58	0.48	0.58	.	.	.
B18	Has he/she had any of the following in the last 12 months: Hypertension or high blood pressure	No	Breast	**0.34**	**0.43**	**0.33**	.	.	.
D13	Which of these devices does he/she uses: hearing aid	use	Breast	1.98	.	.	**3.39**	.	**4.52**
F1	Does he/she regularly go to a senior center	No	Breast	.	.	.	**0.13**	**0.16**	**0.14**
F6	Does he/she usually take a vitamin and/or mineral supplement once a week or more	No	Breast	.	.	.	**0.16**	0.31	0.17
F14	Fish or shellfish?	often	Breast	.	.	.	2.5	.	2.31
F19	Potatoes?	often	Breast	.	.	.	0.42	.	0.45
F21	Other breakfast cereals?	often	Breast	.	.	.	2.48	.	2.36
H12	Does anyone phone or check on him/her regularly just to make sure he/she is all right	no	Breast	2.03	2	2.01	.	.	.
H17	During the past week, did he/she read a book, magazine, or newspaper	no	Breast	**0.11**	0.15	.	.	.	.
H18	During the past week, did he/she work on a hobby, like painting, sewing, or arts and crafts	no	Breast	0.5	**0.39**	0.48	.	.	.
H19	During the past week, did he/she play games such as solitaire or work on puzzles	no	Breast	.	.	.	0.43	.	0.43
H20	During the past week, did he/she listen to records, tapes, (or CDs--in 1994)	no	Breast	.	.	.	0.35	0.5	0.39
H21	During the past month did he/she go to religious services	no	Breast	1.92	1.99	1.99	.	.	.
H22	During the past month did he/she attend a meeting of a civic, religious, professional, or recreational club or org.	no	Breast	.	.	.	0.45	.	0.47
H25	During last two weeks lost his/her appetite / could not eat like he/she usually does	no	Breast	0.38	**0.29**	**0.3**	.	.	.
I2	Is this place part of a building or community intended for older or retired, or disabled persons	no	Breast	.	.	.	**0.28**	.	**0.25**
I4	... ramps	has	Breast	.	.	.	**5.72**	.	**5.1**
I5	… elevators or stair lifts	has	Breast	.	.	.	**6.63**	.	**6.64**
I6	… extra wide doors or hallways	has	Breast	.	.	.	3.31	2.66	**5.58**
I8	… raised toilet	has	Breast	.	.	.	**5.66**	3.15	**6.42**
I10	… extra handrails or grab bars	has	Breast	.	.	.	**5.2**	3.2	**5.87**
I11	… ramps	has	Breast	.	.	.	**8.13**	**5.75**	**16.19**
I12	… elevators or stair lifts	has	Breast	.	.	.	**5.31**	**5.61**	**16**
I13	… extra wide doors or hallways	has	Breast	.	.	.	**6.44**	4.5	**15.55**
I14	… push bars on doors	has	Breast	.	.	.	**7.07**	**14.12**	**30**
I15	… raised toilet	has	Breast	**3.55**	**5.4**	**5.89**	**3.72**	**4.56**	**8.92**
I16	… none	none	Breast	**0.41**	**0.33**	**0.38**	**0.33**	0.45	**0.25**
J6	Is he/she now covered by a private health insurance plan which pays any part of a hospital, doctor's, or surgeon's bill	no	Breast	**0.16**	0.24	0.19	.	.	.
K3	Has he/she been a patient in a hospital overnight or longer, in the last 12 months	no	Breast	.	.	.	**0.29**	**0.21**	**0.23**
K15	...did he/she receive care from a Optometrist in last month	marked	Breast	.	.	.	2.39	**4.35**	.
K18	(Not counting any visits mentioned above)...did he/she receive medical care in a doctor's office	No	Breast	0.52	0.46	0.48	.	.	.
K38	Who will end up paying: Household Members	marked	Breast	3.72	3.32	**5.28**	**7.59**	.	5.07
K39	Who will end up paying: Children of sample person (non-household members)	marked	Breast	.	.	.	**7.55**	.	4.52
K44	In the last month...how many prescription medicines were bought or obtained for him/her	>2+ medicine	Breast	1.9	.	1.9	.	.	.
M4	What are the living quarters?	House, apartment of flat (vs room or mobile home)	Breast	0.42	**0.31**	0.39	.	.	.
N2	Combined Variable: Body Mass Index: Normal	18-25 kg/m**2	Breast	0.56	0.55	0.52	.	.	.
N3	Combined Variable: Body Mass Index: High	higher than 25 kg/m**2	Breast	1.84	1.94	1.99	.	.	.

Table 2 of Electronic Supplemental Material. Relative risks (only significant estimates are shown) of incidental cancer for specific variables, four cancer sites, and two surveys (1994 and 1999). The values of relative risks correspond to outcomes in column 'outcome'. Three used methods are marked as AA (age-adjusted), CT (time follow-up in Cox model), and CA (age follow-up in Cox model).

	Variable	Outcome	Cancer	RR94, AA	RR94, CT	RR94, CA	RR99, AA	RR99, CT	RR99, CA
B1	Rheumatism or arthritis	No	Prostate	.	.	.	**2.41**	2.21	.
B3	Other permanent numbness or stiffness(besides paralysis rheumatism or arthritis)	No	Prostate	2.24	.	.	2.61	.	.
B8	Glaucoma	No	Prostate	15.5	.	.	6.37	.	.
B14	Obesity or he/she is overweight	No	Prostate	.	.	.	0.52	.	0.45
B20	Has he/she had any of the following in the last 12 months: Circulation trouble in his/her arms or legs	No	Prostate	**7.05**	3.65	4.85	**3.36**	.	.
C2	ADL getting in/out of bed	can't	Prostate	0.27	.	.	0.26	.	.
C4	ADL dressing	can't	Prostate	0.09	.	.	0.16	.	.
C5	ADL bathing	can't	Prostate	**0.23**	.	.	**0.16**	.	.
C6	ADL getting to bathroom/using toilet	can't	Prostate	0.09	.	.	**0.09**	.	.
C7	IADL doing heavy work	can't	Prostate	0.43	.	.	0.42	.	.
C10	IADL preparing meals	can't	Prostate	0.17	.	.	**0.1**	.	.
C11	IADL shopping for groceries	can't	Prostate	**0.17**	.	.	**0.23**	.	.
D3	How difficult is it for him/her to: climb one flight of stairs	very difficult/can't do	Prostate	0.34	.	.	**0.08**	.	.
D5	How difficult is it for him/her to: bend to put on socks or stockings	very difficult/can't do	Prostate	0.25	.	.	0.27	.	.
D6	How difficult is it for him/her to: lift a 10-pound package like a bag of groceries and hold it for a few minutes	very difficult/can't do	Prostate	0.45	.	.	0.43	.	.
D11	Does he/she usually see well enough to read ordinary newsprint, with or without glasses or contact lenses	No	Prostate	0.14	.	.	1.92	2.29	2.2
E26	Would he/she say that he/she is physically more, less, or about as active as other persons your age	Less active/same	Prostate	0.54	.	0.56	.	.	.
E29	On a usual day, how much time does he/she spend on light activities	More than 10 min	Prostate	4.97	.	.	1.99	.	1.85
F6	Does he/she usually take a vitamin and/or mineral supplement once a week or more	No	Prostate	.	.	.	0.51	.	0.51
F9	Other dairy products such as yogurt or cheese?	often	Prostate	.	.	.	0.52	0.52	0.53
F10	Eggs?	often	Prostate	.	.	.	0.48	0.46	**0.44**
F17	Pasta such as spaghetti or noodles?	often	Prostate	.	.	.	**0.33**	0.49	**0.34**
F19	Potatoes?	often	Prostate	.	.	.	3.42	.	3.84
F21	Other breakfast cereals?	often	Prostate	.	.	.	1.63	1.83	1.67
G2	Does he/she currently smoke	No	Prostate	0.54	0.44	0.49		.	.
G3	Combined Variable: Intensity of alcohol consumption (at least 1 time a week)	yes	Prostate	**2.17**	2.01	1.79	.	.	.
H1	Compared to other persons the same age, would he/she say that his/her health is…	fair/poor	Prostate	0.45	0.48	0.42	.	.	.
H12	Does anyone phone or check on him/her regularly just to make sure he/she is all right	no	Prostate	**3.21**	2.44	3.34	2.05	.	.
H17	During the past week, did he/she read a book, magazine, or newspaper	no	Prostate	0.34	.	.	**0.18**	.	.
H19	During the past week, did he/she play games such as solitaire or work on puzzles	no	Prostate	.	.	.	2.33	2.87	**3.12**
H22	During the past month did he/she attend a meeting of a civic, religious, professional, or recreational club or org.	no	Prostate	.	.	.	**0.43**	0.58	0.52
I16	… none	none	Prostate	2.74	.	.	2.35	.	.
I20	Is there a conveniently located drug store in the neighborhood	no	Prostate	.	.	.	0.42	.	0.5
K13	...did he/she receive care from a Dentist in last month	marked	Prostate	0.18	0.27	0.18	.	.	.
K18	(Not counting any visits mentioned above)...did he/she receive medical care in a doctor's office	No	Prostate	1.76	.	1.77	**4.52**	2.62	**4.02**
K32	Will insurance, Medicare, Medicaid, or anyone else, including any members of his/her family, end up paying any of the charges for those visits	No	Prostate	.	.	.	2.26	2.03	2.31
K44	In the last month...how many prescription medicines were bought or obtained for him/her	>2+ medicine	Prostate	0.52	0.57	0.58	**0.33**	0.54	0.42
L7	Who was the President just before him	not correct	Prostate	0.16	0.26	0.2	.	.	.
A1	Sex	Male	Lung	**2.86**	2.13	**3.01**	1.73	.	1.8

Table 2 of Electronic Supplemental Material. Relative risks (only significant estimates are shown) of incidental cancer for specific variables, four cancer sites, and two surveys (1994 and 1999). The values of relative risks correspond to outcomes in column 'outcome'. Three used methods are marked as AA (age-adjusted), CT (time follow-up in Cox model), and CA (age follow-up in Cox model).

	Variable	Outcome	Cancer	RR94, AA	RR94, CT	RR94, CA	RR99, AA	RR99, CT	RR99, CA
A2	Race	nonwhite	Lung	2.14	.	1.9	.	.	.
A4	Urban/Rural	city	Lung	1.71	.	1.73	.	.	.
B24	Has he/she had any of the following in the last 12 months: Emphysema	No	Lung	.	.	.	**0.34**	0.32	**0.27**
B27	Has he/she had any of the following in the last 12 months: Other broken bones	No	Lung	**0.35**	.	.	**0.26**	.	0.37
D7	How difficult is it for him/her to: reach above head	very difficult/can't do	Lung	**2.6**	.	.	**3.12**	.	2.58
D10	How difficult is it for him/her to: use fingers to grasp and handle small objects	very difficult/can't do	Lung	**4.53**	.	3.57	.	.	.
D11	Which of these devices does he/she uses: glasses/contact lenses	use	Lung	.	.	.	0.5	.	0.43
D16	Which of these devices does he/she uses: none	not used	Lung	.	.	.	2.19	2.29	2.73
E1	In the past 2 weeks did he/she do: walking for exercise	yes	Lung	0.46	.	0.42	.	.	.
E10	In the past 2 weeks did he/she do: bowling	yes	Lung	5.16	**6.07**	**6.22**	.	.	.
F1	Does he/she regularly go to a senior center	No	Lung	**0.33**	0.45	0.46	.	.	.
F9	Other dairy products such as yogurt or cheese?	often	Lung	.	.	.	0.58	0.53	.
F11	Poultry such as chicken or turkey?	often	Lung	.	.	.	**0.38**	.	0.44
F16	Rice and other grains such as barley or oats?	often	Lung	.	.	.	0.59	.	0.58
F19	Potatoes?	often	Lung	.	.	.	21.3	7.94	17.12
G1	Does he/she currently smoke	No	Lung	**0.21**	**0.24**	**0.2**	0.45	0.38	**0.33**
G4	Combined Variable: Intensity of smoking (at least 1 pack per day)	yes	Lung	**7.39**	**5.77**	**6.51**	**7.04**	**7.19**	**8.3**
H4	Was he/she ever hospitalized for a mental/emotional problem	No	Lung	0.38	0.26	.	.	.	.
H8	Does he/she lose your temper and throw, kick, slam, or destroy things…	not at all	Lung	0.42	**0.3**	0.39	.	.	.
H11	...forget to do important things like eat, take medicine, or pay...bills	no	Lung	.	.	.	**0.3**	0.36	**0.31**
H16	Does he/she have any pets	no	Lung	2.63	2.66	2.96	.	.	.
H17	During the past week, did he/she read a book, magazine, or newspaper	no	Lung	**2.26**	1.95	2.1	0.47	.	.
H21	During the past month did he/she go to religious services	no	Lung	1.76	.	1.74	.	.	.
H23	During last two weeks did not feel like doing the things he/she usually does	no	Lung	0.47	0.45	0.38	.	.	.
H27	Generally speaking, how satisfied is he/she with his/her life as a whole…	not satisfied	Lung	**3.42**	.	3.36	.	.	.
I4	... ramps	has	Lung	2.62	.	2.92	.	.	.
I5	… elevators or stair lifts	has	Lung	**5.48**	.	**4.49**	.	.	.
I18	Is there a toilet or portable toilet conveniently located in the room in which he/she spends most of his/her day	no	Lung	1.97	1.96	1.92	.	.	.
I20	Is there a conveniently located food or grocery store in the neighborhood	no	Lung	.	.	.	**2.57**	2.2	2.28
I21	Is there a conveniently located drug store in the neighborhood	no	Lung	.	.	.	1.76	.	1.82
I22	Is crime a serious problem in this neighborhood	no	Lung	0.49	0.43	0.39	.	.	.
J4	Is he/she now covered by any other public assistance program that pays for health care	no	Lung	0.26	0.16	**0.12**	.	.	.
J6	Is he/she now covered by a private health insurance plan which pays any part of a hospital, doctor's, or surgeon's bill	no	Lung	.	.	.	**3.11**	.	**2.6**
K12	In the last month...did he/she receive care from a dentist, foot doctor, optometrist, or chiropractor	No	Lung	3.51	.	3.5	.	.	.
K14	...did he/she receive care from a Foot doctor in last month	marked	Lung	.	.	.	**3.64**	2.89	**3.63**
K20	Does he/she have a regular source of medical care, like a family doctor, a clinic, or some other medical person or place	No	Lung	**3.42**	2.71	**3.22**	.	.	.
K33	Who will end up paying: Fee for service insurance plans	marked	Lung	0.17	0.18	0.16	0.23	0.33	0.32
K36	Who will end up paying: Medicaid	marked	Lung	**3.41**	.	.	2.23	.	2.91
K37	Who will end up paying: Veterans Administration	marked	Lung	**4.83**	.	3.63	.	.	.

Table 2 of Electronic Supplemental Material. Relative risks (only significant estimates are shown) of incidental cancer for specific variables, four cancer sites, and two surveys (1994 and 1999). The values of relative risks correspond to outcomes in column 'outcome'. Three used methods are marked as AA (age-adjusted), CT (time follow-up in Cox model), and CA (age follow-up in Cox model).

	Variable	Outcome	Cancer	RR94, AA	RR94, CT	RR94, CA	RR99, AA	RR99, CT	RR99, CA
M4	What are the living quarters?	House, apartment of flat (vs room or mobile home)	Lung	.	.	.	**0.35**	.	0.35
N2	Combined Variable: Body Mass Index: Normal	18-25 kg/m**2	Lung	.	.	.	**3.33**	3.08	**3.63**
N3	Combined Variable: Body Mass Index: High	higher than 25 kg/m**2	Lung	.	.	.	0.3	0.34	**0.28**
N5	Besides breakfast lunch and dinner how many other times during the day does he/she eat.	>+1 times	Lung	1.75	.	1.91	.	.	.
A1	Sex	Male	Colon	.	.	.	2.47	.	2.2
A4	Urban/Rural	city	Colon	.	.	.	0.39	.	0.45
B1	Rheumatism or arthritis	No	Colon	1.75	1.86	.	2.21	.	.
B2	Paralysis	No	Colon	**0.16**	0.29	0.22	**0.25**	.	.
B16	Has he/she had any of the following in the last 12 months: A heart attack	No	Colon	.	.	.	**0.25**	.	0.22
B17	Has he/she had any of the following in the last 12 months: Any other hear problem	No	Colon	.	.	.	**0.34**	.	0.3
B21	Has he/she had any of the following in the last 12 months: Pneumonia	No	Colon	0.36	.	0.29	.	.	.
B22	Has he/she had any of the following in the last 12 months: Bronchitis	No	Colon	0.52	.	0.49	.	.	.
D15	Which of these devices does he/she uses: other	use	Colon	**5.31**	.	**4.37**	.	.	.
E1	In the past 2 weeks did he/she do: walking for exercise	yes	Colon	**2.64**	1.83	2.54	.	.	.
E27	On a usual day, how much time does he/she spend on vigorous activities	More than 10 min	Colon	0.34	0.37	0.34	.	.	.
E29	Coffee or tea?	often	Colon	.	.	.	0.42	0.39	**0.27**
G1	Does he/she currently drink any kind of alcoholic beverage, such as beer, wine, or liquor	No	Colon	3.87	2.44	3.95	.	.	.
H17	During the past week, did he/she read a book, magazine, or newspaper	no	Colon	1.89	.	.	**2.78**	.	.
H21	During the past month did he/she go to religious services	no	Colon	0.57	0.56	.	.	.	.
I3	… extra handrails or grab bars	has	Colon	**2.45**	1.88	2.12	.	.	.
I9	… none	none	Colon	0.49	.	0.55	.	.	.
I13	… extra wide doors or hallways	has	Colon	**3.91**	3.51	.	.	.	.
J4	Is he/she now covered by any other public assistance program that pays for health care	no	Colon	.	.	.	0.25	0.24	.
K3	Has he/she been a patient in a hospital overnight or longer, in the last 12 months	no	Colon	.	.	.	0.46	**0.3**	0.38
K4	In the last month...did he/she see a physical therapist, an occupational therapist, or a speech, or a hearing therapist (not counting when he/she was in the hospital)	no	Colon	0.35	0.4	0.34	.	.	.
K35	Who will end up paying: Medicare	marked	Colon	3.4	**4.21**	**5.03**	.	.	.
K15	...did he/she receive care from a Optometrist in last month	marked	Colon	.	.	.	3.04	.	2.8
K32	Will insurance, Medicare, Medicaid, or anyone else, including any members of his/her family, end up paying any of the charges for those visits	No	Colon	2.54	2.27	2.22	.	.	.
L15	What is the name of this city?	not correct	Colon	.	.	.	**10.93**	.	18.73
